# Human adipose tissue-derived stromal cells act as functional pericytes in mice and suppress high-glucose-induced proinflammatory activation of bovine retinal endothelial cells

**DOI:** 10.1007/s00125-018-4713-0

**Published:** 2018-08-27

**Authors:** Ghazaleh Hajmousa, Ewa Przybyt, Frederick Pfister, Genaro A. Paredes-Juarez, Kondaiah Moganti, Stephanie Busch, Jeroen Kuipers, Ingeborg Klaassen, Marja J. A. van Luyn, Guido Krenning, Hans-Peter Hammes, Martin C. Harmsen

**Affiliations:** 1 Department of Pathology and Medical Biology, University Medical Center Groningen, University of Groningen, Hanzeplein 1 (EA11), 9713 GZ Groningen, the Netherlands; 2 5th Medical Department, Medical Faculty Mannheim, University of Heidelberg, Mannheim, Germany; 3 Institute of Transfusion Medicine and Immunology, Medical Faculty Mannheim, University of Heidelberg, Mannheim, Germany; 4 Department of Cell Biology, Molecular Imaging and Electron Microscopy, University Medical Center Groningen, University of Groningen, Groningen, the Netherlands; 5 Ocular Angiogenesis Group, Departments of Ophthalmology and Medical Biology, Academic Medical Center, University of Amsterdam, Amsterdam, the Netherlands

**Keywords:** Adipose tissue-derived stromal cells, Diabetic retinopathy, High glucose, Oxidative stress

## Abstract

**Aims/hypothesis:**

The immunomodulatory capacity of adipose tissue-derived stromal cells (ASCs) is relevant for next-generation cell therapies that aim to reverse tissue dysfunction such as that caused by diabetes. Pericyte dropout from retinal capillaries underlies diabetic retinopathy and the subsequent aberrant angiogenesis.

**Methods:**

We investigated the pericytic function of ASCs after intravitreal injection of ASCs in mice with retinopathy of prematurity as a model for clinical diabetic retinopathy. In addition, ASCs influence their environment by paracrine signalling. For this, we assessed the immunomodulatory capacity of conditioned medium from cultured ASCs (ASC-Cme) on high glucose (HG)-stimulated bovine retinal endothelial cells (BRECs).

**Results:**

ASCs augmented and stabilised retinal angiogenesis and co-localised with capillaries at a pericyte-specific position. This indicates that cultured ASCs exert juxtacrine signalling in retinal microvessels. ASC-Cme alleviated HG-induced oxidative stress and its subsequent upregulation of downstream targets in an NF-κB dependent fashion in cultured BRECs. Functionally, monocyte adhesion to the monolayers of activated BRECs was also decreased by treatment with ASC-Cme and correlated with a decline in expression of adhesion-related genes such as *SELE*, *ICAM1* and *VCAM1*.

**Conclusions/interpretation:**

The ability of ASC-Cme to immunomodulate HG-challenged BRECs is related to the length of time for which ASCs were preconditioned in HG medium. Conditioned medium from ASCs that had been chronically exposed to HG medium was able to normalise the HG-challenged BRECs to normal glucose levels. In contrast, conditioned medium from ASCs that had been exposed to HG medium for a shorter time did not have this effect. Our results show that the manner of HG preconditioning of ASCs dictates their immunoregulatory properties and thus the potential outcome of treatment of diabetic retinopathy.

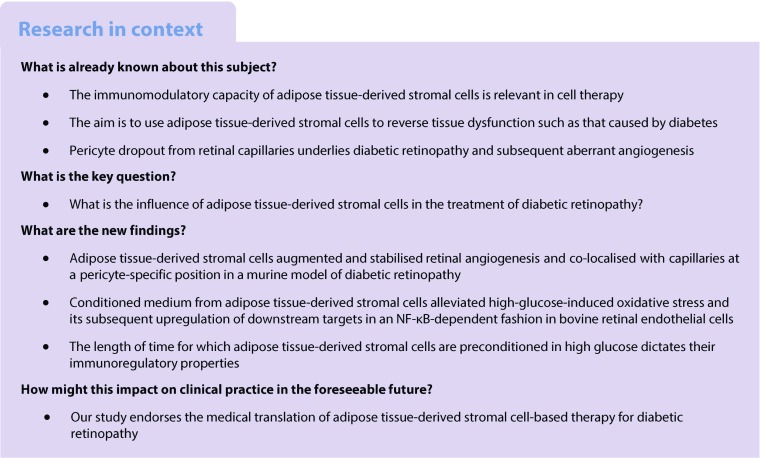



## Introduction

Diabetic retinopathy is the most common microvascular complication of diabetes and remains a leading cause of blindness worldwide [1]. The prevalence of diabetic retinopathy increases with diabetes duration and develops from non-proliferative diabetic retinopathy to proliferative diabetic retinopathy (PDR) and macular oedema [2]. Hyperglycaemia in the retina activates four main biochemical pathway-related changes: (1) polyol pathway flux; (2) accumulation of advanced glycation end-products (AGEs); (3) activation of protein kinase C (PKC); (4) activation of hexosamine pathway flux [3]. Together, these molecular changes induce formation of reactive oxygen species (ROS), which induces impaired retinal blood flow and increased vascular permeability [4]. Retinas or vitreous from diabetic individuals with PDR contain increased levels of proinflammatory mediators including TNF-α, IL-1β [5], IL-6 [6], IL-8 [7] and chemokine (C-C motif) ligand 2 (CCL2) [8, 9]. NF-κB is the main transcription factor that regulates expression of proinflammatory genes. Activation and nuclear translocation of NF-κB promotes forward feedback that augments the proinflammatory state of activated cells [10]. Diabetes is related to the upregulation of cyclooxygenase-2 (COX2) encoded by the prostaglandin-endoperoxide synthase 2 gene (*PTGS2*), both in macro- and microvessels [11]. In retinas of diabetic animals, increased COX2 is followed by increased production of prostaglandin E_2_ (PGE2) [12]. At the onset of diabetic retinopathy, pericyte dropout and the subsequent loss of retinal endothelial cells through apoptosis causes vasoregression. This is the driving force for the pathological angiogenesis of PDR [13]. In the normal retina, pericytes protect and regulate endothelial cell survival and proliferation, vessel integrity and the susceptibility of vascular cells to environmental stimuli [14]. Cell therapy with adipose tissue-derived stromal cells (ASCs) were promising in diabetic animal models. Adipose tissue is easy to acquire and is rich in ASCs. These ASCs are the endogenous mesenchymal stem cells in fat and they harbour a multipotent capacity to differentiate, for example, into adipocytes, chondrocytes and osteoblasts [15]. Recent studies suggested a direct role for ASCs in retinal microvascular support [16]. In rodent models of diabetic retinopathy, ASCs acquired pericytic features, which dampened proliferative angiogenesis [17, 18]. Recently, we showed that the pericytic nature of ASCs depends on neurogenic locus notch homolog protein 2 (NOTCH2)-based juxtacrine interaction between ASCs and endothelial cells [18]. Moreover, ASCs may home in on sites of inflammation [19]. The prime immunosuppressive factors produced by mesenchymal stem cells and ASCs include indoleamine 2,3-dioxygenase [20], PGE2 [21], nitric oxide (NO) [22], IL-10 [23] and antioxidant enzymes such as haem oxygenase-1 [24]. Individually or in combination, these factors also reduce oxidative stress in target cells [25, 26]. We hypothesised that ASCs preconditioned in high glucose (HG) can rescue a dysfunctional retinal endothelium through suppression of the inflammation that was caused by glucose-induced oxidative stress. The aim of this study was to investigate the immunomodulatory paracrine function of ASCs in decreasing the production of endothelial ROS and in the normalisation of dysfunctional endothelial cells in diabetic retinopathy.

## Methods

### Cell isolation and culture

Human subcutaneous adipose tissue samples from healthy individuals (white females aged 25 to <75 years and BMI <30 kg/m^2^) were obtained after liposuction surgery (Bergman Clinics, Zwolle, the Netherlands). Anonymously donated samples were obtained with informed consent as approved by the ethical board of the University Medical Center Groningen, following the guidelines for ‘waste materials’. Lipoaspirates were enzymatically digested to obtain the ASCs, as described in our previous study [27]. The pooled ASCs from passages 3 to 6 were used for experiments. The ASCs were cultured in DMEM (Lonza, Basel, Switzerland) either with normal glucose (NG; 5 mmol/l d-glucose) or HG (30 mmol/l d-glucose) at normoxia (21% O_2_). Continuous HG maintenance of ASCs (more than three passages; ≥21 days) was considered to be chronic HG, while short-term exposure to HG (7 days) was considered to be acute HG. Conditioned medium from ASCs (ASC-Cme) was collected from confluent monolayers after culturing for more than three passages in HG-DMEM. The percentage of FBS was reduced from 10% to 2% (vol./vol.) for 24 h prior to collection [28].

For the co-culture of ASCs and HUVECs, single-cell suspensions of HUVECs were seeded on top of confluent ASCs monolayers or, as a control, on gelatin-coated wells at 10,000 cells/cm^2^ and vascular networks were allowed to form for at least 7 days [29]. Bovine retinal endothelial cells (BRECs) were isolated from freshly-enucleated cow eyes obtained from the slaughterhouse as described previously [30]. First-passage BRECs were used in all experiments. A purity of >99% was routinely achieved in BREC culture, which was checked microscopically, and by immunofluorescence staining of von Willebrand factor (not shown). Confluent monolayers of BRECs were incubated in three different groups (NG-DMEM, HG-DMEM and ASC-Cme) for 7 days to study the effects of each set of conditions.

### Ultrastructural analyses

Co-cultures were fixed in 2% (wt/vol.) glutaraldehyde (Polysciences, Eppelheim, Germany) for 24 h. Samples were post-fixed using osmium tetroxide (Sigma-Aldrich, St. Louis, MO, USA)/potassium ferrocyanide (Sigma-Aldrich) for 30 min. Next, samples were embedded in Epon 812 (SERVA, Heidelberg, Germany) and polymerised at 37°C for 16 h followed by 56°C for 24 h. Thick sections (0.5 μm) were stained with toluidine blue (Sigma-Aldrich). Ultrathin sections (60 nm) were stained with uranyl-acetate (Sigma-Aldrich) in methanol and lead citrate (Sigma-Aldrich). Imaging was performed using a CM100 Biotwin transmission electron microscope (FEI, Eindhoven, the Netherlands).

### Animals and the retinopathy of prematurity model

All animal experiments in this study adhered to the association for research in vision and ophthalmology (ARVO) Statement for the use of animals in ophthalmic and vision research. Male C57BL/6J mice (Charles River, Frankfurt, Germany) were housed with free access to standard chow and water under a 12 h light-dark rhythm. To study the effect of ASCs on hypoxia-driven angiogenesis, newborn mice were subjected to the model of retinopathy of prematurity (ROP) [31]. Mice at postnatal (P) day 7 (P7) were exposed to an atmosphere of 75% oxygen with their nursing mother for 5 days and then returned to room air at P12. Directly after their return to room air, randomly selected mice were intravitreally injected under anaesthesia with either 1 μl of PBS containing approximately 10,000 ASCs (passage 1) or 1 μl of PBS as a control. Eyes were enucleated under deep anaesthesia at P13 for immunofluorescence analysis; at P13 and P19 for quantitative real-time PCR (qPCR) analysis; and at P17 for quantification of neovascularisation. After collection, eyes were immediately fixed in buffered formalin or stored at −80°C for the following analysis.

### Quantification of hypoxia-driven neovascularisation

Neovascularisation in retinas was assessed in paraffin sections of P17 animals injected at P12 with ASCs or a control. To this end, sections (6 μm) were stained with periodic acid–Schiff’s reagent (Sigma-Aldrich). Nuclei of neovessels at the vitreous side of the inner limiting membrane of the retinas were counted as described previously [32].

### Assessment of ASCs in vivo

Whole-mount retinas from P13 animals were permeabilised with 0.5% (wt/vol.) Triton-X100 at room temperature for 1 h. Overnight staining was with FITC/TRITC-labelled isolectin-B4 (1:50; Sigma-Aldrich) at 4°C. After PBS washes, retinas were flat-mounted in glycerol and micrographs were obtained with a fluorescence microscope (Lectin-FITC/ASC-Dil red staining; Leica BMR, Bensheim, Germany) or a confocal microscope (Lectin-TRITC/ASC-EGFP staining; Leica TCS SP2 confocal microscope, Leica, Wetzlar, Germany). ASCs presence was revealed through their pre-injection label, which was either CM-DiI-red (ThermoFisher, Waltham, MA, USA) or enhanced green fluorescent protein (EGFP) lentiviral tag.

#### Gene expression analysis

Total RNA was extracted from ASCs and BRECs in TRIzol reagent (Life Technologies, Carlsbad, CA, USA) following the manufacturer’s protocol. Retinas were isolated from frozen eyes of ROP and control mice at P13 (*n* = 5) and ROP mice at P19 in the presence of ASC injection or PBS (*n* = 5). Afterwards, 1 μg of total RNA from each sample was reverse transcribed using the First Strand cDNA Synthesis Kit (Fermentas, Vilnius, Lithuania) according to the manufacturer’s instructions. The cDNA equivalent of 10 ng RNA was used for amplification in 384-well plates in a TaqMan ABI 7900HT thermal cycler (Applied Biosystems, Foster City, CA, USA) in a final reaction volume of 10 μl containing 5 μl SYBR Green Universal PCR Master Mix (BioRad, Hercules, CA, USA) and 6 μmol/l primer mix (forward and reverse). The cycle threshold (C_t_) values were normalised to *GAPDH/ACTB* as a reference gene using the ΔΔC_t_ method [33].

#### Assessment of cell viability

Viability was assessed using the Apoptosis & Necrosis Kit (Promokine, Heidelberg, Germany) as recommended in the manufacturer’s instructions. In short, BRECs were incubated with 5 μl fluorescein-conjugated annexin V (a marker of apoptosis) and 5 μl ethidium homodimer III (at a concentration of 2 × 10^6^ cells/ml) at room temperature for 15 min. Fluorescence was recorded on a BD FACSCalibur (BD Biosciences, Franklin Lakes, NJ, USA) within 1 h of staining.

#### Quantification of ROS

Cellular ROS production was determined using the dye 2′,7′-dichlorofluorescein diacetate (DCFH-DA, Sigma-Aldrich). Two-electron oxidation of DCFH-DA results in the formation of a fluorescent product, dichlorofluorescein (DCF) [34]. Experimental cells were suspended in 20 μmol/l DCFH-DA in the dark at 37°C for 15 min. The general ROS scavenger *N*-acetyl-l-cysteine (NAC, 10 mmol/l, Sigma-Aldrich) and H_2_O_2_ (3 μmol/l, Merck Millipore, Darmstadt, Germany) were used as negative and positive controls, respectively. Samples were analysed directly using a FACSCalibur within 15 min of staining.

#### Screening for immune stimulation

The supernatant of HG-stimulated BRECs cultured with or without ASC-Cme and the control group (BRECs in NG) were collected after 7 days. To assess the immunogenicity of these samples, THP1-XBlue-MD2-CD14 cells (InvivoGen, Toulouse, France) were used as described previously [35]. THP1-XBlue-MD2-CD14 cells were plated, then each well was stimulated with samples of supernatants and cultured overnight at 37°C in 5% CO_2_. Lipopolysaccharide (LPS, 10 μg/ml, Sigma-Aldrich) was used as a positive control. Production of SEAP in the supernatant was quantified using QUANTI-Blue (InvivoGen). An aliquot of QUANTI-Blue (200 μl) was dispensed into a new flat-bottomed 96-well plate with 20 μl of supernatant from the stimulated cell-lines for 45 min at 37°C. Secreted embryonic alkaline phosphatase (SEAP) activity, representing activation of NF-κB, was then measured at a wavelength of 650 nm on a VersaMax microplate reader (Molecular Devices, Biberach an der Riss, Germany) [35].

#### ELISA

Culture medium was collected from different experimental conditions. The concentrations of PGE2 and CCL2 in the medium were quantified, respectively, with the Prostaglandin E2 Human ELISA Kit (ThermoFisher) and the Human CCL2/MCP-1 DuoSet ELISA (R&D Systems, Minneapolis, MN, USA) according to the manufacturer’s protocol. LPS (200 ng/ml) and TNF-α (50 ng/ml, Sigma-Aldrich) were used as positive controls. The COX2 inhibitor celecoxib (10 mmol/l, Sigma-Aldrich) was used to inhibit PGE2 production. Results were normalised to the number of cells in each experimental condition and presented in pg/ml as fold change relative to their respective experimental controls.

#### Monocyte adhesion assay

One of the cardinal steps of inflammation is the infiltration of immune cells such as monocytes across the endothelial cell layer [36]. THP-1 monocytes were stained using the Vybrant CFDA SE Cell Tracer Kit (ThermoFisher) according to the manufacturer’s instructions. Treated groups of BRECs (under HG with or without ASC-Cme), a positive-control group (pre-incubated with medium containing TNF-α [10 ng/ml] for 8 h) and a control group (NG) were plated at a density of 5 × 10^3^ cells/cm^2^ in standard 96-well culture plates and allowed to adhere at 37°C for 4 h. Labelled THP-1 cells (2 × 10^5^ cells/ml) were added to each well. After incubation for 1 h, wells were washed to remove non-adhered cells. Fluorescence of adherent cells was recorded on a Varioskan spectrofluorometer (Thermo Scientific, Waltham, MA, USA) at excitation/emission = 492/520 nm [37].

#### Scratch wound healing assay

BRECs in three different groups (NG, HG and ASC-Cme) were cultured until they reached confluency. The straight, width-limited scratch was made in all the wells, simulating a wound. The recovery of both wound edges was recorded simultaneously using the Solamere Nipkow confocal live cell imaging microscope (Solamere Technology Group, Salt Lake City, UT, USA) for 30 h. The percentage of covered area between the edges was analysed by ImageJ 1.8.0-172 software (imagej.nih.gov/ij/download/).

#### Statistical analysis

Data are expressed as mean ± SEM and relative to vehicle controls of at least three independent experiments in triplicate. Statistical evaluation was performed using unpaired *t* tests and ANOVA followed by Bonferroni post hoc analysis. *p* values <0.05 were considered statistically significant.

## Results

### Ultrastructure of ASC-induced vascular networks

**Fig. 1:**
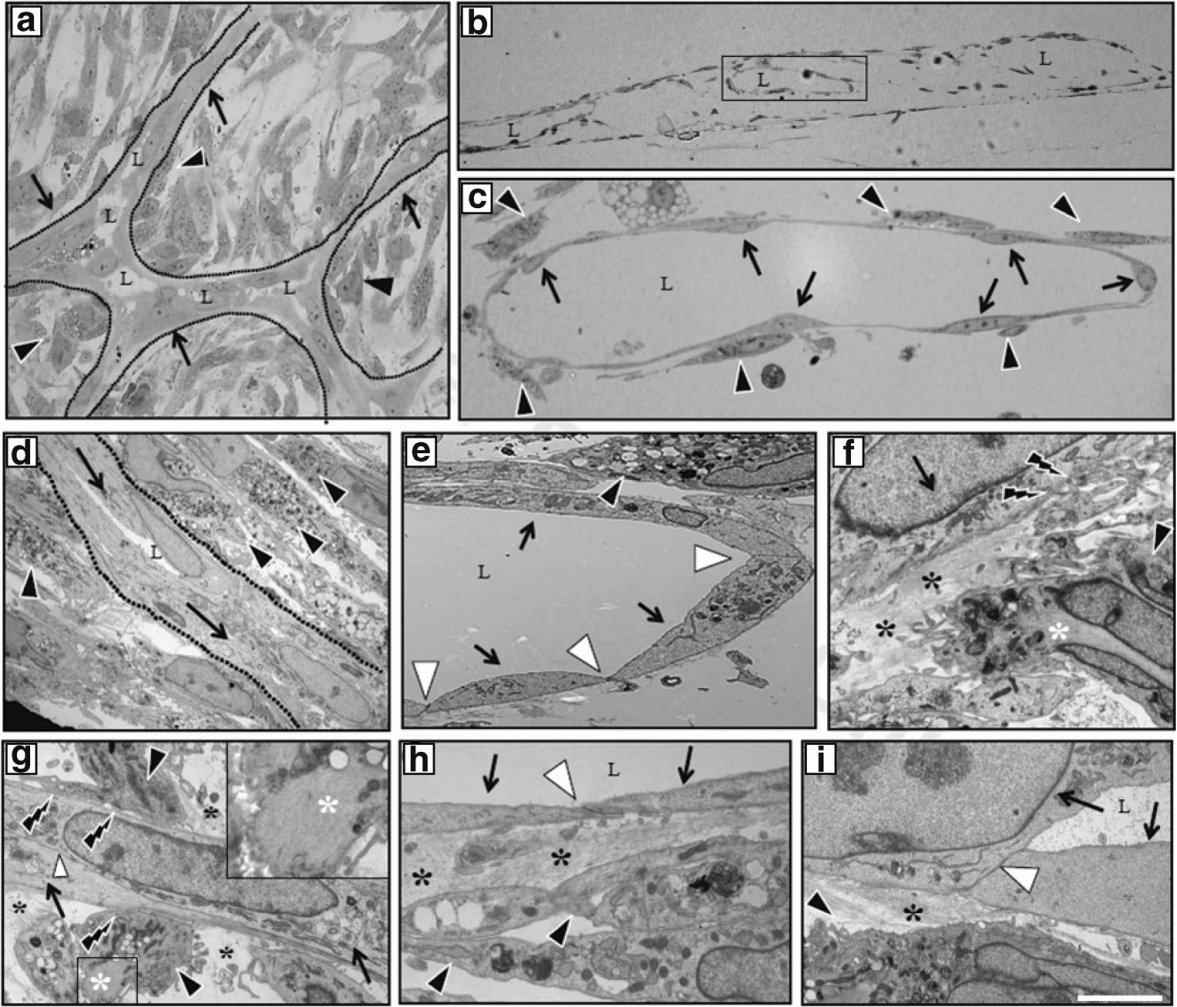
Ultrastructure of ASC-induced vascular network. HUVECs were seeded on confluent monolayers of ASCs. After 5 days, 0.5 μm sections were stained with toluidine blue and analysed by light microscopy, and 60 nm sections of glutaraldehyde-fixed and plastic-embedded co-cultures were analysed by transmission electron microscopy. (**a**–**c**) Representative light micrographs: (**a**) Planar, parallel section (i.e. the top view of the culture) showing the formation of a vascular network (arrows, endothelial cells) demarcated by the dotted lines. Lumens (L) have formed, which are aligned by ASCs (black arrowheads) in close contact. (**b**) Cross section of the lumen-containing 3D vascular structures in between (interrupted) layers of ASCs. (**c**) Enlargement of a vascular structure with several aligned ASCs (arrows, endothelial cells; black arrowheads, ASCs). (**d–i**) Transmission electron micrographs of the vascular structures: (**d**) a vascular structure consisting of endothelial cells (arrows) and lumen is depicted by the dotted lines with surrounding ASCs (black arrowheads). (**e**) Specific cell–cell connections with tight junctions between endothelial cells (white arrowheads), with lumen formation on top of the ASCs. (**f**) ASCs deposit extracellular matrix (black asterisks), which forms a basement membrane-like structure between the endothelial cells and the ASCs. (**f**, **g**) Peg-and-socket connections are shown by the lightning symbols, and the inset in (**g**) shows intracellular filaments (white asterisks), indicative of contractility, similar to smooth muscle cells, i.e. hinting at the maturation of ASCs to pericytes. (**h**, **i**) Detailed views of the endothelial cell–cell connections and basal membrane formation around the endothelial cells, i.e. the vascular structure with connected ASCs. Scale bar, 5 μm. Lumen (L); endothelial cells (arrows); ASCs (black arrowheads); endothelial cell–cell connections (white arrowheads); extracellular matrix formation (black asterisks); intracellular filaments representative for smooth muscle cell phenotype (white asterisks); peg-and-socket connections of ASCs with endothelial cells (lightning symbols)

The 0.5 μm cross-sections showed that interconnected laminar structures had been formed by co-cultures of endothelial cells on ASC monolayers at day 5 and subsequent days (Fig. 1a). Transmission electron micrographs of longitudinal sections showed the build-up of 3D structures that comprised endothelial-cell-derived (Fig. 1c) vessel-like structures with a lumen (Fig. 1a–c), surrounded and tightly aligned by ASCs (Fig. 1c). At high magnification, these vessel-like structures appeared as intermittent structures between the ASCs (Fig. 1d). The vascular networks formed were reminiscent of genuine vessels with respect to the slanted intercellular junctions between endothelial cells (Fig. 1e, h, i). Peg-and-socket processes [38, 39] that extended from ASC-derived pericytes to endothelial cells had also formed (Fig. 1f, g), as well as an extracellular matrix-based membrane between pericytes and endothelial cells (Fig. 1f, i). ASC-derived pericytes, i.e. those cells in close contact with endothelial cells, had lost their typically abundant vesicular contents compared with more distal ASCs (Fig. 1d). These ultrastructural results indicate that ASCs promoted formation of vascular networks by endothelial cells and that ASCs had acquired a functional pericytic phenotype in vitro (Fig. 1g).

### ASCs stabilise hypoxia-driven angiogenesis but also engraft at pericytic sites in the animal model of ROP

**Fig. 2:**
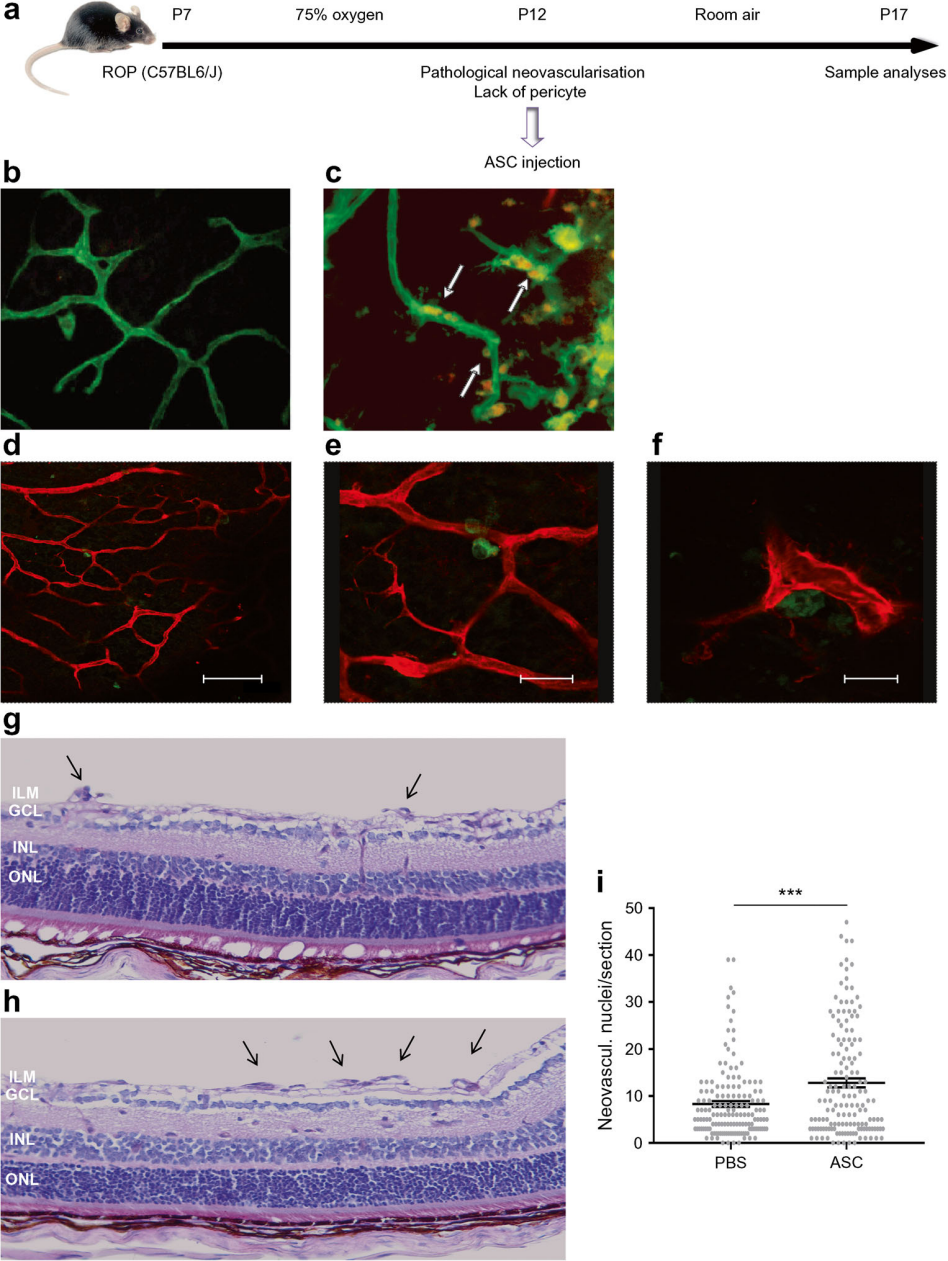
ASCs enhance hypoxia-driven angiogenesis in the ROP mouse model. (**a**) Scheme of the ROP model. Mouse pups were exposed to hyperoxia (75% O_2_) from P7 to P12 and subsequently transferred to room air (21% O_2_). This causes hypoxia at room air that results in extensive retinal neovascularisation at P17. ASCs were injected into the vitreous at P12 to evaluate their influence on hypoxia-driven neovascularisation. (**b**, **c**) Representative micrographs of CM-DiI-labelled ASCs (red, white arrows) co-localising with the endothelial layer (lectin, green); magnification × 20. (**d**–**f**) Micrographs of EGFP-tagged ASCs (green) co-localising with the endothelial layer (lectin, red) in the pericytic position. The scale bars in (**d**–**f**) are 100 μm, 25 μm and 15 μm, respectively. (**g**, **h**) Histological analysis of the effects of ASC injection on hypoxia-induced retinal neovascularisation; magnification × 20. Neovascularisation was assessed histologically by counting the endothelial cell nuclei anterior to the inner limiting membrane. (**g**) Histological features of retinal neovascularisation in the control group. (**h**) Histological features of retinal neovascularisation in ASC-injected mice. Intravitreal injection of ASCs in the ROP mouse model increased the number of neovascular tufts extending into the vitreous (black arrows). (**i**) Hypoxia-driven neovascularisation in the retinas was enhanced by 54% in animals injected with ASCs (PBS vs ASC, 8.3 ± 0.62 vs 12.8 ± 0.96). The graph shows the mean number of neovascularisation nuclei per section per animal, ***p < 0.001. GCL, ganglion cell layer; ILM, inner limiting membrane; INL, inner nuclear layer; neovascul., neovascularised; ONL, outer nuclear layer

ASCs were injected intravitreally at P12 in the eyes of ROP-model mice (Fig. 2a). At P13, injected ASCs were present in angiogenic sprouts and had attached to endothelial cells of maturating capillaries at a pericyte-like position (arrows, Fig. 2b, c). Further analyses showed that dye-labelled-ASCs (green, Fig. 2d–f) had aligned and attached to maturing intraretinal capillaries (red, Fig. 2d–f) at pericyte-specific perivascular positions and in contact with the endothelium.

Quantification of hypoxia-induced retinal neovascularisation at P17 showed that injection of ASCs in the animal models of ROP increased neovascularisation by 54% (Fig. 2g–i; PBS vs ASCs, 8.3 ± 0.62 vs 12.8 ± 0.96).

### ASCs modulate the inflamed ROP micro-environment

**Fig. 3:**
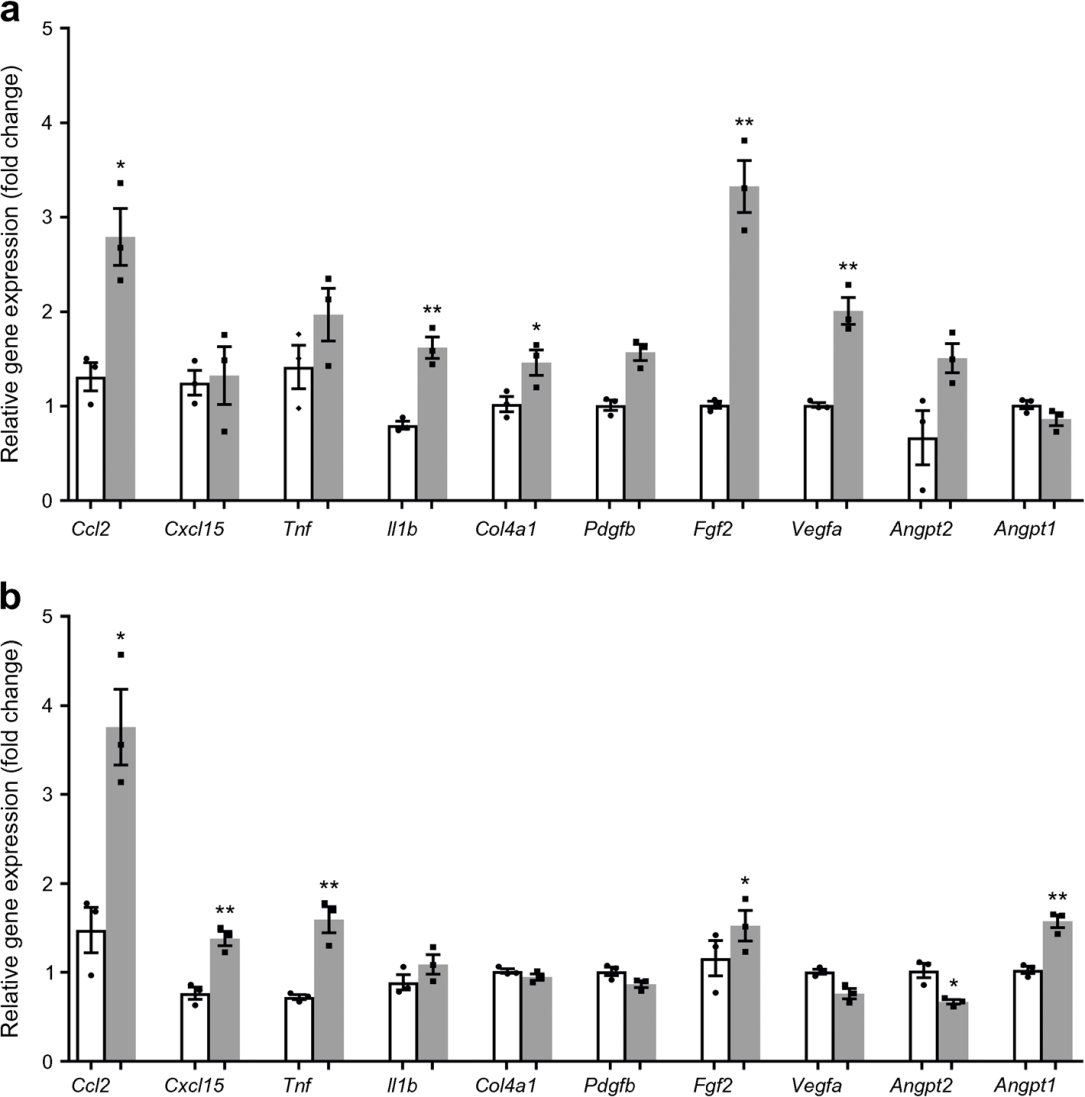
ASCs modulate the ROP micro-environment. Gene expression analyses normalised to *Gapdh* of (**a**) dissected ROP retinas at P13 (grey bars) compared with the control retinas at P13 (white bars). The expression levels of *Vegfa*, *Fgf2*, and *Col4a1* were increased. In addition, an inflammatory response was induced, as measured by increased expression of *Il1b* and *Ccl2*. (**b**) To assess the ASC-guided changes to the ROP micro-environment at P19, gene expression in ROP retinas of eyes with ASC injection (grey bars) were compared with controls (white bars). We observed increased expression of *Angpt1* and *Fgf2*, and decreased expression of *Angpt2* in ROP retinas of eyes with ASC injection. The expression of *Vegfa*, *Pdgfb* and *Col4a1* was similar to controls. The inflammatory response was modulated by normalised *Il1b* expression, while the expression of *Tnf*, *Cxcl15* and *Ccl2* was increased. *p < 0.05, **p < 0.01 vs control retinas. Graphs show the means ± SEM from retinas of five animals in each group; experiments were performed in triplicate

ROP and control retinas (P13) were assessed by qPCR. At P13, i.e. 24 h after returning the animals from hyperoxia to ambient oxygen, *Angpt1* (encoding angiopoietin [ANGPT] 1) expression had slightly decreased in ROP retinas, while *Angpt2* had slightly increased (although neither significantly) compared with the non-ROP controls (Fig. 3a). This was accompanied by increased expression of angiogenesis-related genes i.e. *Vegfa* (encoding vascular endothelial growth factor [VEGF] A; 2.0-fold change), *Fgf2* (3.2-fold change), and *Col4a1* (1.4-fold change) (Fig. 3a). The analysis showed increased expression of proinflammatory genes i.e. *Il1b* (2.0-fold change) and *Ccl2* (2.1-fold change) compared with P13 controls. *Cxcl15* (mouse orthologue of human *CXCL8*) and *Tnf* changed non-significantly (Fig. 3a). Comparison of ROP retinas with and without ASC intervention showed that injection of ASCs at P12 caused a 1.5-fold increase of *Angpt1* and a 0.6-fold decrease of *Angpt2*, suggesting induction of vascular quiescence. Injection of ASCs into the ROP eyes caused increased expression of inflammatory genes such as *Tnf*, *Cxcl15* and *Ccl2* by 2.2-, 1.8- and 2.5-fold, respectively. A slight decrease was observed in the expression of proangiogenic *Vegfa* and a 1.3-fold increase of *Fgf2* was observed in ROP at P19 with ASCs, compared with the control ROP at P19 without ASCs (Fig. 3b). No differences in the expression of *Pdgfb*, *Col4a1* and *Il1b* were detected.

### Chronic or acute exposure to HG influences ASC gene expression

**Fig. 4:**
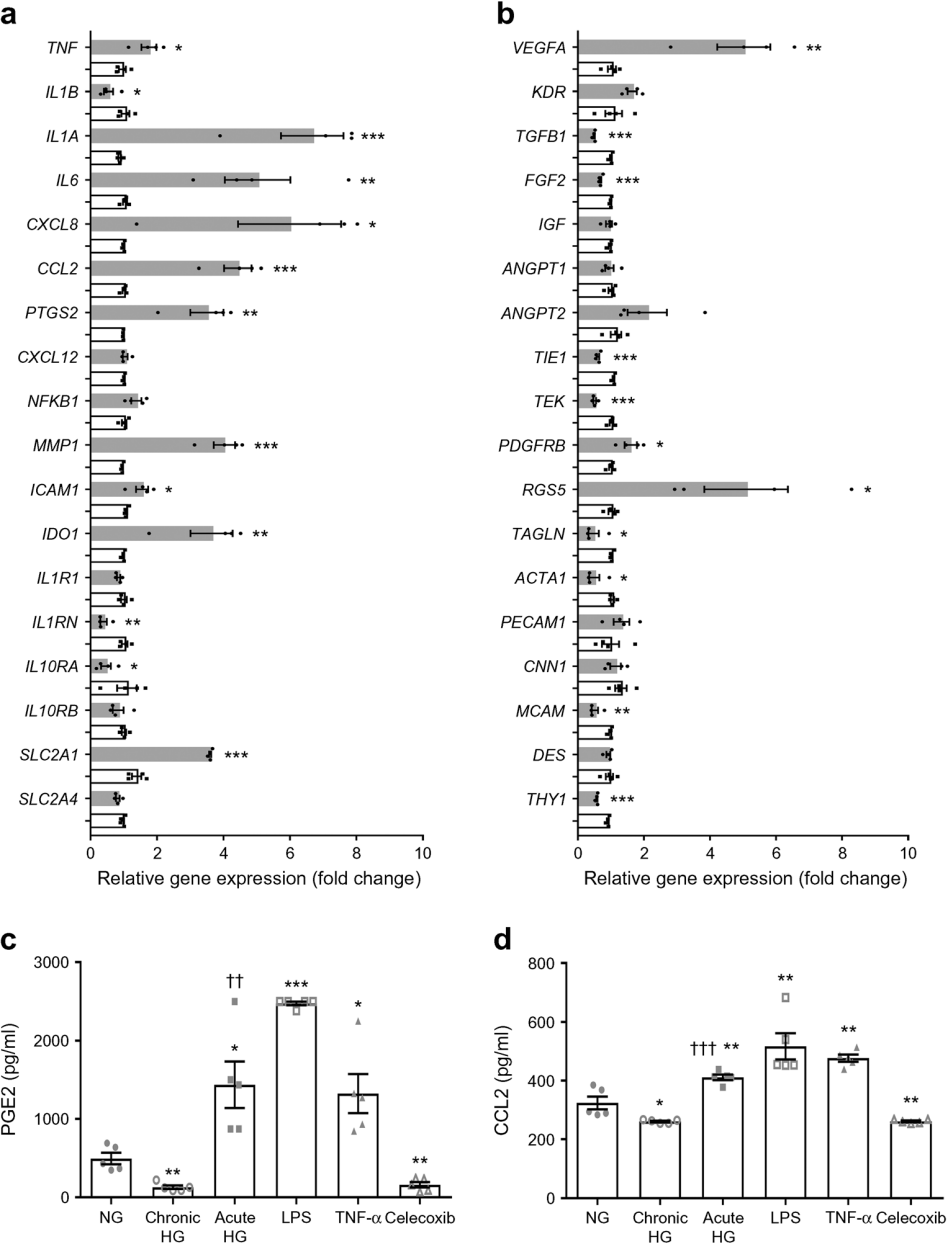
Anti-inflammatory and anti-apoptotic effects of ASCs depend on chronic HG preconditioning. (**a**, **b**) Expression of 36 genes, normalised to *ACTB*, in ASCs after acute exposure to HG (7 days) or chronic HG (more than 21 days maintenance in HG) compared with NG-exposed controls. Grey bars, acute HG exposure; white bars, chronic HG exposure. Graphs represent data ± SEM from *n* = 4 independent experiments. *p < 0.05, **p < 0.01, ***p < 0.001 vs NG-exposed control. (**c**, **d**) The inflammatory response in HG conditions was induced in ASCs and measured by ELISA to detect PGE2 and CCL2 in ASC-Cme. LPS and TNF-α were used as positive-stimulated controls. Celecoxib (10 mmol/l) was used as an inhibitor of COX2 in acute-HG-treated ASCs. Graphs show mean ± SEM from *n* = 5 independent experiments. *p < 0.05, **p < 0.01, ***p < 0.001 vs NG control; ††*p* < 0.01, †††*p* < 0.001 vs chronic HG. Genes encode the following proteins: *TNF*, tumour necrosis factor; *IL1B*, IL-1β; *IL1A*, IL-1α; *IL6*, IL-6; *CXCL8*, C-X-C motif chemokine ligand 8 (also known as IL-8); *CCL2*, chemokine (C-C motif) ligand 2; *PTGS2*, prostaglandin-endoperoxide synthase 2; *CXCL12*, C-X-C motif chemokine 12; *NFKB1*, NF-κB subunit 1; *MMP1*, matrix metallopeptidase 1; *ICAM1*, intercellular adhesion molecule 1; *IDO1*, indoleamine 2,3-dioxygenase 1; *IL1R1*, IL-1 receptor type 1; *IL1RN*, IL-1 receptor antagonist; *IL10RA*, IL-10 receptor subunit α; *IL10RB*, IL-10 receptor subunit β; *SLC2A1*, solute carrier family 2 member 1 (also known as GLUT1); *SLC2A4*, solute carrier family 2 member 4 (also known as GLUT4); *VEGFA*, vascular endothelial growth factor A; *KDR*, kinase insert domain receptor/VEGF receptor 2; *TGFB1*, TGF-β1; *FGF2*, fibroblast growth factor 2; *IGF*, IGF; *ANGPT1*, angiopoietin 1; *ANGPT2*, angiopoietin 2; *TIE1*, tyrosine kinase with immunoglobulin like and EGF like domains 1; *TEK*, TEK receptor tyrosine kinase (also known as Tie2); *PDGFRB*, platelet derived growth factor receptor β; *RGS5*, regulator of G-protein signalling 5; *TAGLN*, transgelin; *ACTA1*, actin α1; *PECAM1*, platelet/endothelial cell adhesion molecule 1 (skeletal muscle); *CNN1*, calponin 1; *MCAM*, melanoma cell adhesion molecule (also known as CD146); *DES*, desmin; *THY1*, Thy-1 cell surface antigen (also known as CD90)

The expression of 36 relevant genes was measured in ASCs after acute exposure to HG (i.e. maintained in NG medium, followed by 7 days in HG medium) or chronic exposure to HG (i.e. always maintained in HG medium and for more than 21 days). Data are presented as the fold change compared with (chronic) maintenance in NG medium (Fig. 4a, b). The acute exposure of ASCs to HG upregulated the proinflammatory genes *TNF* (1.8-fold change), *IL1A* (6.0-fold change), *IL6* (4.5-fold change), *CCL2* (4.3-fold change) and *CXCL8* (6.0-fold change); as well as the proangiogenic genes *VEGFA* (4.8-fold change), *ANGPT2* (1.8-fold change) and *MMP1* (4.2-fold change). Expression of the angiopoietin receptors *TIE1* and TEK (also known as *TIE2*) was downregulated (0.52-fold and 0.47-fold change, respectively). The glucose transporter *SLC2A1* (encoding GLUT1) was upregulated 2.5-fold. Interestingly, acute HG exposure induced a 4.9-fold change in the mesenchymal pericyte marker *RGS5*, while expression of other mesenchymal markers such as *ACTA1*, *TAGLN* and *PDGFRB* changed only marginally. Endothelial marker *PECAM1* was also upregulated (1.3-fold change). The expression of two immunoregulatory genes, *PTGS2* (encoding COX2) and *IDO1*, were upregulated (3.4-fold and 3.6-fold change, respectively). The expression of all genes in ASCs was similar upon culture under chronic HG and chronic NG conditions, which indicated that ASCs adapt to different glucose concentrations in the culture medium during long-term culture. The inflammatory response after acute exposure to HG resulted in an increased release of PGE2 (11.0-fold change) and CCL2 (1.6-fold change) by ASCs, compared with chronic exposure to HG (Fig. 4c, d). Interestingly, chronic exposure to HG medium slightly decreased PGE2 secretion (0.3-fold change) and secretion of CCL2 (0.8-fold change) compared with controls maintained in NG. LPS and TNF-α both induced the secretion of PGE2 and CCL2, while celecoxib inhibited the production of PGE2 completely and had less effect on secretion of CCL2 in acute-HG-stimulated ASCs.

### The antioxidant role of ASC-Cme abrogates NF-κB activation and promotes cell viability of BRECs

**Fig. 5:**
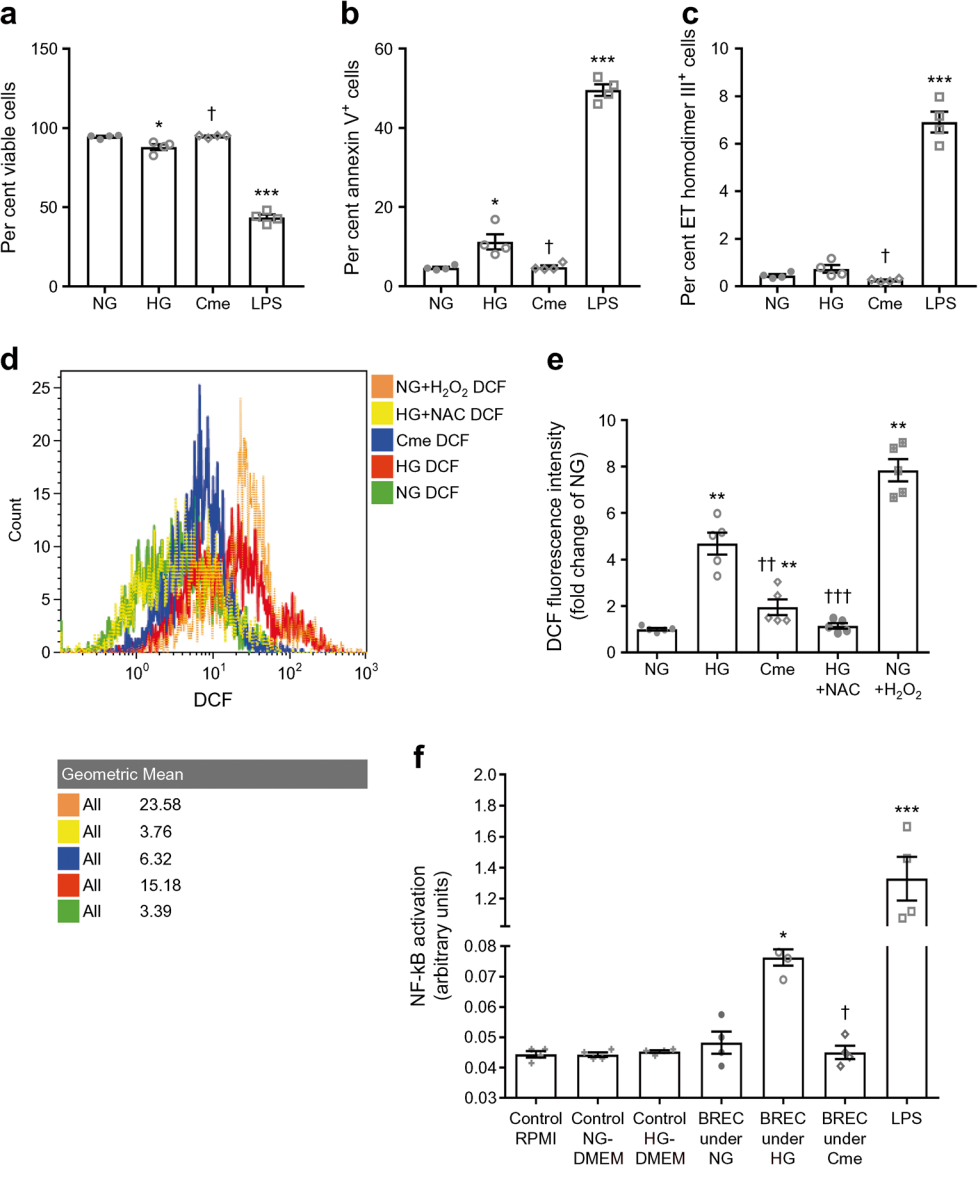
The antioxidant role of ASC-Cme, combined with declining NF-κB activation, promotes cell viability of HG-challenged BRECs. (**a**) ASC-Cme promotes BREC viability following HG-induced apoptosis (viability ∼88% vs 94.6%, HG vs NG; 88% vs 93.9%, HG vs Cme). (**b**) HG-induced apoptosis was normalised by ASC-Cme (annexin V-positive cells ∼11.2% vs 4.6%, HG vs NG; 11.2% vs 4.8%, HG vs Cme). (**c**) Necrosis (ethidium [ET] homodimer lll-positive cells ∼0.73% vs 0.45%, HG vs NG; 0.73% vs 0.25% HG vs Cme). (**a**–**c**) LPS was used as a positive control. (**d**) The histogram shows the representative increase of fluorescence intensity (by DCF) after exposure to HG or H_2_O_2_ control compared with NG or ASC-Cme and NAC treatment. (**e**) HG induces ROS in BRECs. Total cellular ROS production was measured by DCF. ASC-Cme suppressed ROS production in the presence of HG, compared with HG alone. The ROS inhibitor NAC and H_2_O_2_ were used as negative and positive controls, respectively. (**f**) NF-κB activation by conditioned BREC medium in THP1-XBlue-MD2-CD14 cells, and mediated by ASC-Cme. NF-κB activation was significantly higher in cells treated with BREC conditioned medium under HG conditions (BREC under HG), compared with unstimulated control. This response was almost absent when cells were treated with ASC-Cme alongside BREC conditioned medium under HG conditions (BREC under Cme). LPS was used as a positive control for THP1-XBlue-MD2-CD14 cells and induced NF-κB activation. RPMI-1640 medium, NG-DMEM and HG-DMEM were used as controls, which had no effect on activation of THP1-XBlue-MD2-CD14 cells. Absorbance values were plotted to express NF-κB activation with arbitrary units. *p < 0.05, **p < 0.01, ***p < 0.001 vs NG control; †*p* < 0.05, ††*p* < 0.01, †††*p* < 0.001 vs HG. Values are mean ± SEM (*n* = 4 in **a**–**c**, **f** and *n* = 5 in **e**). Cme, ASC-Cme

Culture of BRECs under HG conditions for 7 days induced cell death (viability ∼88% vs 94.6% for HG vs NG, Fig. 5a). Apoptosis was increased (annexin V-positive cells ∼11.2% vs 4.6% for HG vs NG, Fig. 5b), while necrosis had also increased (ethidium homodimer lll-positive cells ∼0.73% vs 0.45% for HG vs NG, Fig. 5c). Simultaneous treatment of HG-stimulated BRECs with ASC-Cme (i.e. conditioned medium from ASCs chronically cultured in HG medium) normalised viability (∼93.9% vs 88% for ASC-Cme vs HG, Fig. 5a) and suppressed apoptosis (∼4.8% vs 11.2% for ASC-Cme vs HG, Fig. 5b) and necrosis (∼0.25% vs 0.73% for ASC-Cme vs HG, Fig. 5c) of the BRECs.

Exposure of BRECs to HG increased intracellular ROS production (4.6-fold change compared with NG controls, Fig. 5e), while ASC-Cme abrogated this (0.4-fold change, Fig. 5e). This was comparable to the NAC effect on HG-induced BRECs (0.25-fold change, Fig. 5e).

The monocytic NF-κB reporter (SEAP) cell line THP1-XBlue-MD2-CD14 was used to assess the proinflammatory capacity of conditioned medium obtained from BRECs that were co-treated with HG and ASC-Cme. The strongest activation of NF-κB occurred after stimulation with LPS (29.0-fold change, Fig. 5f), while neither NG nor HG medium (DMEM) had any influence. Compared with NG controls, the activation of monocyte-expressed NF-κB increased (1.4-fold change) upon exposure to conditioned medium from HG-stimulated BRECs. In contrast, NF-κB activation was normalised to NG control levels (0.6-fold change) by conditioned medium from BRECs that were HG induced and simultaneously treated with ASC-Cme.

### ASC-Cme downregulates principal inflammatory factors in HG-challenged BRECs

**Fig. 6:**
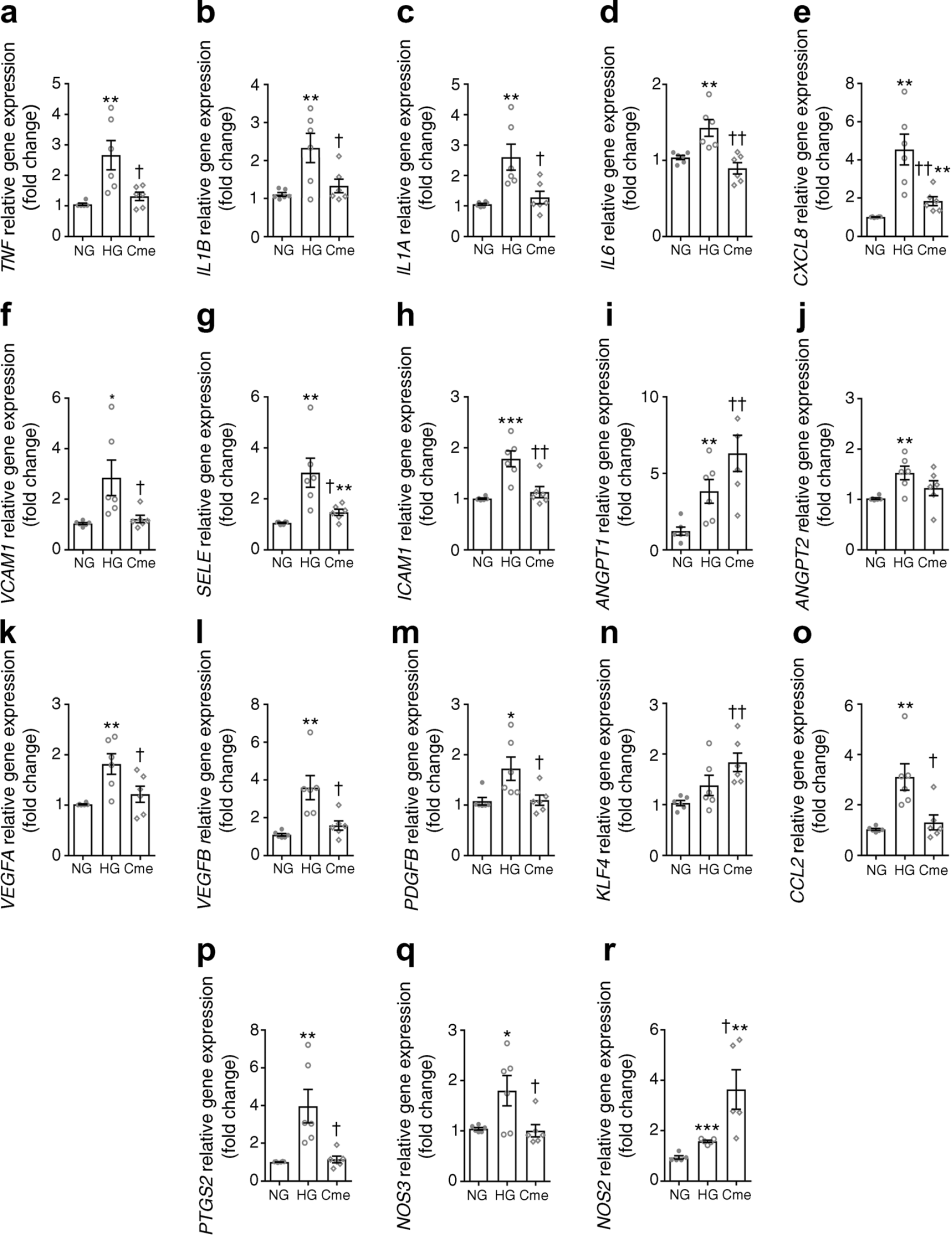
ASC-Cme downmodulates the main inflammatory genes in HG-challenged BRECs, compared with NG-treated controls. (**a**–**r**) HG upregulated the expression of the main genes related to inflammation (excluding *KLF4*, **n**). The upregulation of *TNF*, *IL1B*, *IL1A*, *IL6*, *CXCL8*, *VCAM1*, *SELE*, *ICAM1*, *VEGFA*, *VEGFB*, *PDGFB*, *CCL2*, *PTGS2*, and *NOS3* was significantly modulated (reduced) by ASC-Cme. ASC-Cme upregulated the gene expression of *ANGPT1*, *KLF4* and *NOS2*. Values are mean ± SEM (*n* = 6). *p < 0.05, **p < 0.01, ***p < 0.001 vs NG control; †*p* < 0.05, ††*p* < 0.01 vs HG

HG stimulation of BRECs for 7 days upregulated the gene expression of the relevant proinflammatory genes *TNF*, *IL1B*, *IL1A*, *IL6*, *CXCL8* and *CCL2*, as well as leucocyte adhesion-related genes *SELE*, *ICAM1* and *VCAM1.* In addition, the proangiogenic genes *ANGPT1*, *ANGPT2*, *VEGFA*, *VEGFB* and *PDGFB*, and the endothelial NO synthases, *NOS2* and *NOS3*, were upregulated, as well as *PTGS2*. The upregulation of these genes was normalised to NG control level in ASC-Cme-treated HG-challenged BRECs. Only *KLF4* did not change after HG challenge of BRECs. Interestingly, expression of both *KLF4* and *NOS2* were upregulated in ASC-Cme-treated HG-challenged BRECs. The expression of *ANGPT1*, a vessel quiescence-associated factor, remained increased upon ASC-Cme treatment of HG-challenged BRECs (Fig. 6a–r).

**Fig. 7:**
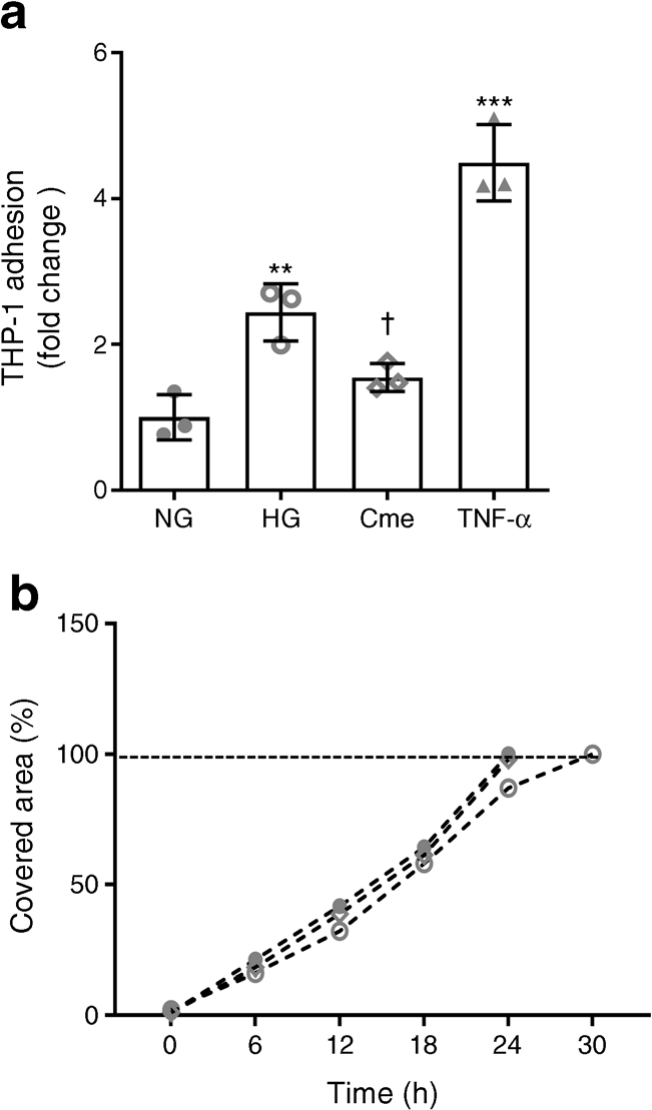
(**a**) THP-1 cell adhesion to BRECs exposed to HG, with and without ASC-Cme. The intensity of fluorescence of adherent THP-1 cells (mean ± SEM, *n* = 3, fold change of NG-treated control) in resting or activated BRECs was measured. A significant decrease in adhered THP-1 cells occurred after treatment of HG-challenged BRECs with ASC-Cme, compared with HG treatment alone. (**b**) Scratch wound healing assay to study interaction in the BREC monolayer under NG, HG and ASC-Cme conditions*.* The percentage of covered area between the wound edges was analysed. The percentage signifies the remaining gap size 30 h after making the scratches, compared with the initial gap size. The gap width decreased in a similar pattern in all three groups (NG, closed circles; HG, open circles; ASC-Cme, open diamond) and was not significantly delayed in HG conditions. Values are mean ± SEM. **p < 0.01, ***p < 0.001 vs NG control; †*p* < 0.05 vs HG

As expected, the proinflammatory condition of HG-challenged BRECs (Fig. 6a–r) also increased their adhesiveness to THP-1 monocytes (2.4-fold change, Fig. 7a), while treatment with ASC-Cme normalised monocytic adhesion to NG levels (0.6-fold change). Treatment of BRECs with TNF-α promoted the strongest adhesion of monocytes (4.5-fold change, Fig. 7a).

Wound closure of BRECs cultured in NG medium, after HG challenge or treatment with ASC-Cme during HG challenge, was similar over a measurement period of 30 h, after which time full closure occurred (Fig. 7b).

## Discussion

The main results of our studies are that human ASCs stimulate retinal angiogenesis in ROP mice via acquisition of a pericytic position and function. This was confirmed by ultrastructural analyses showing that in vitro ASCs promote vascular network formation of endothelial cells and stabilise these networks via a pericytic function. Extracellular matrix was deposited between ASC-derived pericytes and endothelial cells in vitro, while typical peg-and-socket connections were also formed [38, 39]. Acute exposure of ASCs to HG caused a proinflammatory and proangiogenic activation compared with ASCs that were propagated chronically in HG. In fact, conditioned medium from ASCs that had been chronically exposed to HG reduced ROS and proinflammatory activation of BRECs challenged with HG. Activated genes were reduced to normal expression levels, while endothelial protective genes such as *KLF4* were upregulated. The immunomodulatory properties of ASCs [40–42] may vary upon passaging in culture [41]. Early passages of ASCs express markers, e.g. ligands that stimulate cells of the adaptive immune system such as MHCII, CD80 and CD86, which are lost upon passaging [41, 43]. Our results show that ASC injection into ROP mice, a model of pathological angiogenesis in PDR [44], led to an increased expression of inflammatory genes such as *Tnf*, *Cxcl15* and *Ccl2*, which might relate to the early passaging of ASCs. The balance of *ANGPT1* and *ANGPT2* and the expression of *VEGFA* are among the most important regulators of vascular development, maturation and maintenance [45]. Reduced expression of pericyte-derived ANGPT1, and concomitant upregulation of its antagonist ANGPT2 and increased VEGFA levels induce pericyte dropout, vessel destabilisation and sprouting angiogenesis [46]. In ROP retinas of ASC-treated mice, expression of *Angpt1* and *Fgf2* were increased, while levels of *Angpt2* and *Vegfa* were reduced. Injected ASCs adhered to neovessels at a pericytic position. This corroborates the role of NOTCH2 in the juxtracrine function of ASCs [18]. Earlier work from our group showed that under acute HG culturing, ASCs largely maintain their pericytic role as an endothelial supporting cell, despite an upregulated ROS production accompanied by increased apoptosis in ASCs [29].

**Fig. 8:**
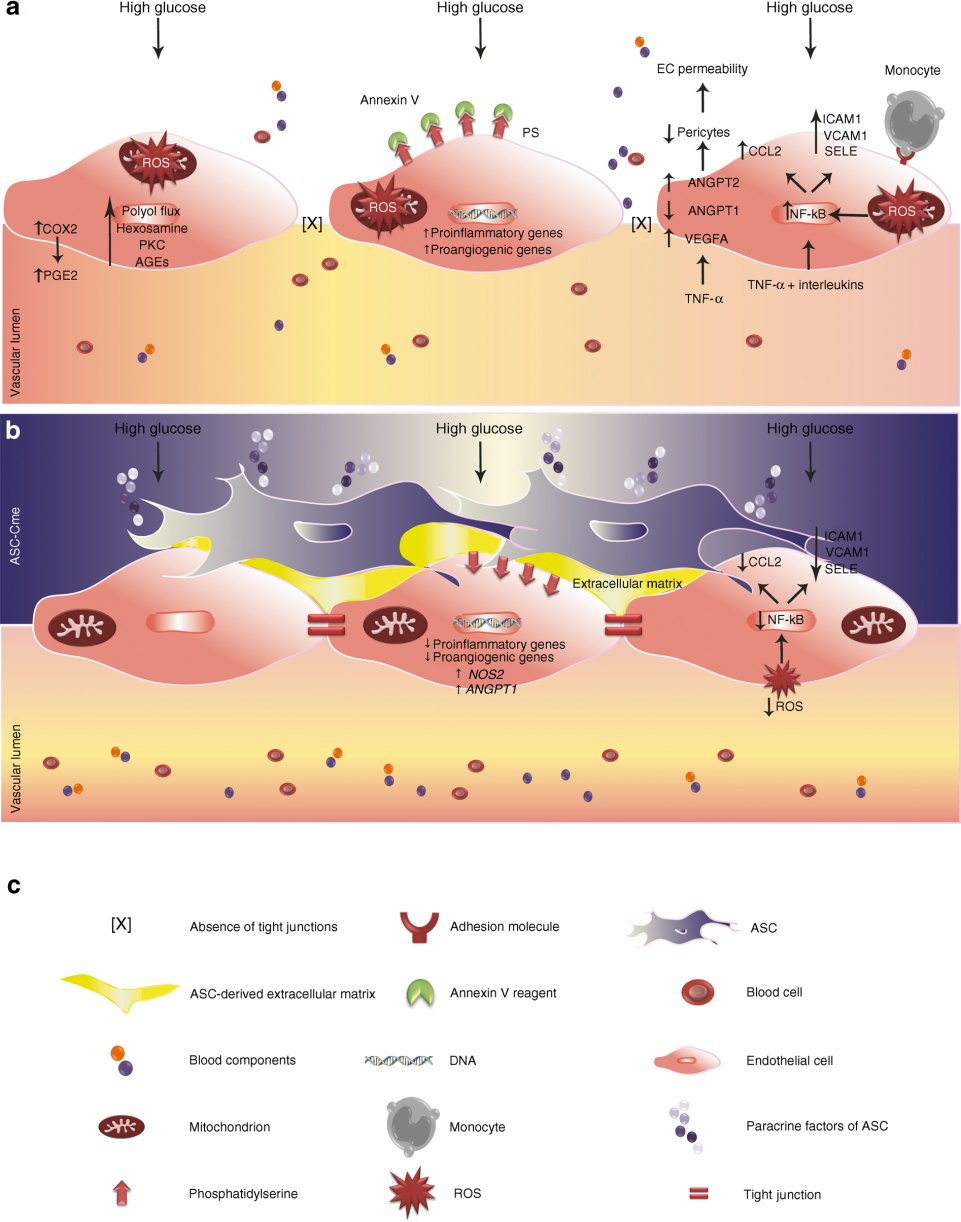
Therapeutic actions of ASCs in the normalisation of HG-challenged BRECs: a roadmap to the treatment of diabetic retinopathy. (**a**) BRECS under HG conditions and (**b**) with the addition of ASC-Cme. This model was corroborated in HG-activated BRECs by the suppression of ROS production by ASC-Cme, together with a reduction in monocyte adhesion and improved cell survival. ASCs preconditioned in HG can rescue dysfunctional retinal endothelium through suppression of the inflammatory and proangiogenic genes induced by glucose-induced oxidative stress, and can stabilise their vascular networks via pericytic function. EC, endothelial cell; ICAM1, intercellular adhesion molecule 1; PS, phosphatidylserine; SELE, selectin E; VCAM1, vascular cell adhesion molecule

In general, most papers use DMEM as constitutive culture medium to propagate ASCs. This medium contains 25 mmol/l d-glucose, which is, in fact, similar to hyperglycaemic conditions in vivo. A main finding of our study was that chronic exposure of ASCs to HG medium normalised gene expression to levels seen under NG conditions. Interestingly, in comparison to chronic propagation in HG, ASCs that were shifted from NG-DMEM to HG-DMEM for 7 days had upregulated gene expression of proinflammatory cytokines and chemokines, but also of proangiogenic factors. Moreover, secretion of PGE2 and CCL2 were also strongly increased. COX2 is responsible for the diabetes-induced retinal PGE2 production, and inhibition of COX2 inhibited the diabetes-induced upregulation of retinal VEGF, which links COX2 expression to angiogenesis in diabetic retinopathy [12, 47, 48]. PGE2 activates the extracellular signal-regulated kinases 1 and 2 (ERK1/2) and thus promotes angiogenesis through endothelial upregulation of the secretion of e.g. VEGF, chemokines and activation of cell cycle genes [49, 50]. Thus, the acute HG exposure of ASCs likely renders these cells in a proangiogenic state. Their upregulated expression of proinflammatory genes likely also activates endothelial cells. Yet the exposure of retinal endothelial cells to HG alone activated NF-κB and increased expression of downstream *IL1B*, *VEGFA*, *TNF* and *ICAM1* to name a few of the upregulated main proinflammatory genes. ASC-Cme suppressed these proinflammatory genes such as *PTGS2* in BRECs plus *VEGFA* as a proangiogenic gene. Acute HG exposure of ASCs would induce endothelial dysfunction rather than counteract it. However, the continuous propagation of ASCs in HG medium not only suppresses their proinflammatory status, but also augments their capacity to normalise endothelial cell function. This was corroborated in HG-activated BRECs by the suppression, by ASC-Cme, of ROS production, the reduction of monocyte adhesion and improved cell survival (Fig. 8). Part of the immunosuppression resides in the suppression of endothelial ROS production by ASC-secreted factors, because ROS functions, via activation of TAK1, as an activator of NF-κB. Taken together, our results show that HG preconditioning of ASCs potentially improves their therapeutic efficacy as compared with short-term exposure to environmental conditions such as HG.

## Data Availability

Data supporting the conclusions of this article are included within the article and are available from the corresponding author on reasonable request.

## Abbreviations

ANGPT: Angiopoietin
ASC: Adipose tissue-derived stromal cell
ASC-Cme: Conditioned medium from ASCs
BREC: Bovine retinal endothelial cell
CCL2: Chemokine (C-C motif) ligand 2
COX2: Cyclooxygenase-2
DCF: Dichlorofluorescein
DCFH-DA: 2′,7′-Dichlorofluorescein diacetate
HG: High glucose
LPS: Lipopolysaccharide
NAC: *N*-acetyl-l-cysteine
NG: Normal glucose
NO: Nitric oxide
NOTCH2: Neurogenic locus notch homolog protein 2
P: Postnatal day
PDR: Proliferative diabetic retinopathy
PGE2: Prostaglandin E_2_
PKC: Protein kinase C
qPCR: Quantitative real-time PCR
ROP: Retinopathy of prematurity
ROS: Reactive oxygen species
SEAP: Secreted embryonic alkaline phosphatase
VEGF: Vascular endothelial growth factor
